# Some Remarks on Insanity; Its Classification and Nomenclature

**Published:** 1912-09

**Authors:** F. St. John Bullen

**Affiliations:** Late Assistant Medical Officer and Pathologist, West Riding Asylum


### SOME REMARKS ON INSANITY; ITS CLASSIFICATION AND NOMENCLATURE.

BY

F. ST. JOHN BULLEN, M.R.C.S. Eng.,

*Late Assistant Medical Officer and Pathologist, West Riding Asylum.*

---

CLASSIFICATION is needed for the purpose of isolating diseases or disorders from each other where an independent existence can be determined. Although it is more difficult to draw sharp distinctions between forms of mental than of bodily disease, yet it is possible, from amongst the manifold symptoms of insanity, to isolate some naturally cohesive groupings without assuming that such syndromes are in themselves discrete entities.

Schemes of classification have been attempted for over half a century; the earlier ones necessarily largely metaphysical, then clinical, the later ones clinico-pathological. At first only leading divisions were made, with added variations recognised as modifications due to personal idiosyncrasy or environment. As acquaintance with the influences of hereditary predisposition, causation, epochs in development, physiological crises, increased, so the scope of classification was enlarged, and gradually an extensive, perhaps rather artificial collection of supposed symptom-groups was arrived at. For practical purposes each of these would carry certain expectancies in course and outcome, in casual liabilities and treatment; but as the subdivisions increased it became evident that the complexity rather clouded than cleared the survey. In fact, any attempt at a too strict demarcation of forms of insanity must fail, for no two cases of mental disorder can exactly correspond. Each case is an entity expressing a specific reaction of the mind to some particular influence, and groups must vary as the individual cases. A certain aggregate of symptoms, too, may have any of various causes, or the same cause produce various symptoms. Again, some ruling disposition, such as suspicion or pride, may exist as a background in mild, acute or chronic insanity, giving, so to speak, an atmosphere in common to maladies of quite a distinct nature.

It must not be supposed that those responsible for elaborate schemes of causal or symptomatological kind have meant to create artificial varieties, but that their object has been to embrace many cases possessing certain common characteristics into collections, in each of which should be represented a leading cause, symptom or outlook giving a distinctive aspect. Thus such groups might be differentiated from others showing a similar type of reaction, but with another origin. Despite the greater knowledge of the modern school of psychiatry, the tendency of late has been to revert to broad groupings and to the elimination of merely symptomatic, diathetic and fanciful arrangements. At the same time, efforts have been made to combine various states seemingly independent, or, on the other hand, suffering from casual or false attachments. Very few conditions in insanity can fulfil the requirements of a typical disease in which there should be a reasonably constant relationship between the organic lesions and the symptoms. The variety of mental phenomena is great, the morbid expressions of them and the idiosyncrasies of individuals inexhaustible; thus, most insane conditions are disorders, and their peculiarities determined by varying causes or dispositions.

The earlier of the more recent schemes of classification comprised the vesanic or functional disorders, the diathetic and sympathetic, the toxic. Later on were added groups based on developmental and involutional influences, partial insanities, associated psychoses. At that time distinctions were not attempted between toxic states of internal or external origin. The recognition of the hereditary degenerative psychoses was a most important event, and into these were incorporated most of the existing monomanias.

A still further elaboration has resulted from greater knowledge of causation and ascertained pathological conditions. As for moral insanity, it may be elided from a modern classification as an entity; in its various phases it is included under the hereditary degeneracies as constitutional immorality, sexual perversions, etc. The true degeneracies, as partitioned from the constitutional tendency to the arousal on excitation of a latent psychoneurosis, imply a veritable decay of stock, a predestination to insanity. Such include the hallucinated deliria, hysteria, chronic neurasthenia with obsessions and impulses, hereditary intellectual weakness, dementia precox and paranoia (according to some authorities).

Before a satisfactory table of classification can be set forth, it is necessary to discuss more or less recent modifications or changes in nomenclature, as also the altered constitution of certain syndromes by limitation or extension, and by the incorporation or exclusion of other forms of disorder.

It is especially requisite to survey the group of mental disturbances formerly included under the title of Chronic Delusional Insanity, and now known in part as systematised delirium, in part as hereditarily degenerate insanity; in either case with or without the existence of hallucinations. Here will be found many of the old monomanias. We may at once premise that it is not yet possible to take some of these forms out of the region of debate, so far as the question of their individual independence is concerned, and we think too much segregation has been attempted; in reality, the forms overlap. It is of greater importance to comprehend the general characters underlying these delusional disorders and their modifications than to seek an unfeasible separation into distinct disorders.

The main features in the isolation of certain forms from amongst the mass of delusional insanity may be briefly stated, They are the presence or absence of influential hallucinations, the regular or the episodic development of the malady and its delusions, the basis of growth, the presence or absence of an established order of symptoms in evolution, the number, interdependence, transience or stability of the delusions, and the existence or not of notable degeneracy in the person or family. Accordingly the establishment of two important groups has been essayed. Firstly, hereditarily degenerate forms, including insanity of unsystematised evolution with hallucinations (or the hallucinatory delirium of degenerates); this is sometimes, and questionably well, called paranoidal delirium; also systematised insanity without hallucinations, called by the French school "*délire de revendication*," or "mania of persecuted persecutors." Secondly, psychoses with congenital predisposition, but not with actual degeneracy evident; these are paranoia or interpretive insanity and Magnan's *délire chronique* with hallucinations. The essential in all these forms is the so-called paranoical diathesis, in which exists the nucleus of all kinds of delusional insanity, whether persecuted, revengeful, exalted or hypochondriacal in type. The variations are occasioned by the relative strength and quality of the morbid heredity, and to some extent by the exciting cause where this can be said to exist. Inasmuch as the condition indicates chronic mental disequilibrium by its obsessions, impulses, phobias, impotences, youthful outbreaks, rapid onset and advance, hurried variations in mood and preposterous ideas, hallucinations, quickly evolved exaltation and altered personality, it points to a preponderance of real degeneracy. In so far as the growth of the malady is slow, developing in a recognised course, with stable and connected delusions and with pseudo-hallucinations or without any hallucinations at all, with comparatively preserved lucidity and long-postponement of dementia, in so far may the disorder be viewed as the deferred expression of an anomaly of development, a perversion rather than disintegration of personality, a constitutionally distorted but yet serial mode of thought.

Morbid heredity is regarded as the most influential factor in all these states, but between the extremes of true paranoia and the hallucinatory delirium of degenerates there are marked distinctions—the order of mind inherited and the mental attitude are opposed in many particulars. The characterising features of the degenerate psychoses are revealed even in the comparatively mild states of psychasthenia—vacillations, obsessions, struggles of desire with a restraining personality, a coercive invitation to actions of an irrelevant kind from the outside, a state of mental conflict pursued for vain ends. The self is undermined by internal dissension. In the more pronounced degree of insanity of degenerates these features are exaggerated into sudden storms of mood and conduct, hasty maturation or episodic progress of the malady, readily-evolved delirious ideas, sudden exaltation, a medley of delusions, rapid change in or dissolution of the personality. Hallucinations are responsible for adding confusion and impulsiveness to the mental state. In paranoia, although the primary false idea may be suddenly realised, development from it is slow, steady and durable, the conduct logically determined, the struggle waged with an antagonistic environment. The ego is eventually completely enthralled by the masterful conceptions, and acts with precision and vigour. The disposition is often calm, reflective, mystical, complex, patiently researchful, and the paranoiac seeks to alter his environment in conformity with his changed self and to enforce his views upon it. The degenerate is tempted to act by some fancied defect in his surroundings, needing urgent redress, often with a sense of personal slighting or wrong, and his reaction is marked by its haste, intemperate passion, and unbalanced reasoning. The paranoiac always shows a steady exalted egoism, but his pride is poisoned by distrust and the primitive mysticism and romanticism, essentials of his character, continually expose him to imaginary hostile influences. Notwithstanding this apparently emotional atmosphere, the prominent anomaly is intellectual ; incorrigible misinterpretation occurs in certain lines of thought. Degenerative insanity is, on the contrary, a quasi-maniacal condition, a sensory delirium,manifested rather in turmoil of ideas and action than in the laborious weaving of fresh delusions.

The various transitional forms between the extreme members of the delusional group just described have not at present settled limitations and nomenclature. We think that the term paranoia should be restricted to the type above indicated, which develops with system, without ruling hallucinations, and in which restriction of mental life rather than dementia occurs ; that systematised insanity, which is based upon or largely influenced by hallucinations, is classically illustrated in Magnan's well-known syndrome, *délire chronique* ; that amongst the hereditarily degenerate forms we can recognise one of a relatively unsystematic evolution nurtured on hallucination, and which can be termed the hallucinatory delirium of degenerates, and also one more regularly developed, not dependent on hallucinations, and which is identifiable with the French *délire de revendication.*

It is evident that no hard and fast line can be drawn between these different forms ; modifications will be graduated in accordance with morbid predisposition and other factors, which in proportion to degree and kind prepare for the calm, logical and interpretive state, or for the more excitable, erratic, impulsive and hallucinated one. In all it is observable that grave mental reductions are implied by the fundamental ego-centricism ; but whilst in paranoia the personality remains coherent though altered, in the markedly degenerative insanities it suffers disintegration ; permeated by false relations and erroneous conclusions, it is transformed and dominated by them. The extent of the morbid egoism, or again, the dissolution of the self when recognisable, is the best estimate of the relative seriousness of each form as met with, together with the partial or general limitations apparent in the intellectual sphere and the dissociation in thought. The more impaired the relation between subjective and objective consciousness and that of intra-psychic states, the more obtrusive become the derangements of cœnesthesis and the dominating egoism; so also results the destructive rather than the constructive type of the new personality or main delusive concept.

Just as several differing states have been collected under paranoical psychoses, so under the term dementia precox a grouping of apparently varied conditions has been attempted. No psychosis has received more attention and criticism, and though as a term it is freely adopted at the present, there is far from being agreement as to its significance and limitations. It is an example of the difficulties that attend the enforced separation of a definite disorder from the multiple states which result from the diverse factors of hereditary taint, environment, epoch of life, acquired habits and intercurrent ailments. Let it not be thought that there is any wholly novel disorder here established; most of the phases were familiar to acute observers, from Griesinger onwards. The synthesis of several apparently independent disturbances of mind into one all-embracing, though exceedingly complex syndrome, is however the work of Kraepelin. It remains to be seen whether his labours have been exerted to a clear and persistent end. This author and his followers would gather into dementia precox all the old adolescent insanities, all the maniacal and melancholic attacks separable from manic-depressive insanity, most of the stupors and catatonic states. The English acceptation of the title simply implies the almost inevitable result, and not the elaborate symptomatology that precedes. There are many phases in the disorder, but it more especially represents those cases of insanity arising at the adolescent period which from marked degenerative tendencies, prolonged physical drain, mental exhaustion, or moral depravation of masturbatic habits, break down gravely and quickly. But here let it be said that it is unjustifiable to include all outbreaks at the adolescent period as inevitable dementia precox; there are many cases of mania or melancholia that recover at this time, and which may be viewed as isolated attacks in a predisposed person, or the first of a periodic and recoverable series. In any case, however, the mental disorder possesses the features of extravagant, boastful, puerile, inconsequent conduct, or agitated and impulsive melancholia, with more or less confusion and often a background of suspicion. These states, in the more strongly hereditary cases, often pass into stupor with but partial recovery, or into dementia. All cases of stupor at adolescence, again, must not be assumed a phase of dementia precox, inasmuch as some forms following on acute emotional or confusional disorders of other kind are recoverable. In these there is an emotional background, inferential at the time and often revealed later, which is foreign to the apathy of dementia precox. In such status attonitus consciousness is not wholly in abeyance, more or less coherent delusions and hallucinations may be present, prompting negative resistance. The condition is rather a suspension than an abolition of intelligence and active expression—one analogous to the hypnotic lethargy, and frequently to be traced to mental shock, exhaustion or toxæmia.

Another phase of dementia precox, although less typical, is one of simple lethargy. It was of this doubtless that Griesinger wrote as follows (though without making any separation from the group of primary dementias): "There are states where the perversion of affections and apathy appear the chief elements . . . . a state mainly acquired by onanists and drunkards." Now and then will be found an adolescent of seemingly average intelligence who has passed into a purely apathetic condition, careless as to his present state and future career, indifferent to his family, yet excepting a certain mild hypochondriacal self-absorption, without other manifest change. The habit of self-abuse or excess in alcohol is by no means invariably traceable. This passive form is at the best only partially recoverable; notable gaps in intelligence, permanent feebleness of will, or unstable and freakish conduct will remain. Such cases no doubt were formerly grouped under primary dementia, and their salient features—gradual mental extinction with marked emotional apathy—were aptly enough so termed.

Taking dementia precox as a whole, the most characteristic qualities are the sectional intellectual deterioration and emotional lethargy. The defect appears less of perception and memory than of serial and relative thought. Although there is a general mental enfeeblement, it is the discontinuity, the irregularity, the inchoate character of the intellectual operations that is the striking feature. As paranoia has been described as representative of a reversion to the mental traits of a primitive race, so precocious dementia, in its hebephrenic stage especially, is a return towards what Bevan Lewis has described as "the excito-motor irritability of infancy, with its jerky, spasmodic, ill-directed movements wanting in object, in power, in co-ordination, in skill." The behaviour of these cases is marked by its haphazard, irresponsible, flighty or reflex nature; in such there would seem to be a hazy familiarity with the complex environment and a child-like inability for immediate adjustment to this. The association system, perhaps imperfectly evolved or organised, appears at fault, and this intra-psychic severance confers the aspect of discontinuity and inappositeness which is characteristic. There is also observable the divorce of will from reason; motives to action seem as the stray promptings of a low delirium; conduct in relation to environment is often the height of inappropriativeness in reaction; hence results negativism, mutism or blind impulse. Reduced at times to an almost purely automatic level, the patient's behaviour when excited is monkey-like, embracing mimicries of words or gestures, antics, stereotyped and senseless performances, purposeless destructiveness. Circumstances are only known to them to be misconstrued or resisted. In the milder forms the same characters are perceptible—perversions of mood and conduct, waywardness, inconsequent or bizarre behaviour, suspiciousness, vague hypochondriacism, and the utterance of crude, silly or disconnected statements. In most other forms of mental decay there is some recognition of anomalous thought and conduct by the patient himself, but in dementia precox the person seems unconscious of or indifferent to the change, and his behaviour only expresses the aggravated puerility and self-satisfaction which indicates the serious impairment of consciousness. The stolidity and emotional indifference with a low suspicion are also opposed to the energies, the assertive and acquisitive tendencies of the normal adolescent male, or to the reflectiveness and sensitive receptivity of the female.

Many of the states already described, which include inertia, stereotypism, immovability, negative resistance have been formerly grouped as katatonia; this last has been merged into dementia precox. Now to this already complex syndrome Kraepelin has proposed the addition of yet another group, that is unsystematised insanity of degenerative kind with or without hallucinations, but of course ending early in dementia. Cases, then, of dementia precox which do not become arrested or pursue a course of mere fatuity, but which behave as chronic mania with fragmentary delusions and hallucinations, would be classed as the paranoidal demented; the delusions unsystematised, the hallucinations erratic, the conduct disorganised. Why such condition, whether part of precocious dementia or not, should have received the title of dementia paranoides is not evident; the essential character of paranoia or interpretive insanity is the absence of confusing hallucinations and unsystematised delusions. It is a disorder particularly methodical, slow, logical, with an unalterable bias in many if not all of the patient's intellectual operations. Prognosis where premature dementia is suspected is of high importance, for a positive forecast is one of appalling moment. There has to be borne in mind the possibility of occasional and recoverable attacks of acute confusion; of a primary attack of recurrent mania or melancholia with stupor-katatonia; of youthful progressive paralysis; of alcoholic delirium or episodic excitement and confusion in degenerate persons; transient persecutory or ambitious ideas in the congenitally feeble-minded. There is little doubt as to the strong inherited neurotic taint in dementia precox; but whilst some claim that dementia precox is most typical when occurring in young adults of average intelligence, others would have it to be grafted, not seldom, on a mild congenital weakness. A certain proportion would probably belong to the class of inborn moral defect, especially complicated by masturbation, and brought into active prominence at adolescence. Bevan Lewis, writing of such cases, remarks: "Prior to the mental commotion at this epoch, these moral imbeciles have, it may be, shown an aptitude for learning, a brightness of intelligence proportionate to their age." Lewis regarded the stuporose, depressed and hallucinatory forms of adolescent insanity as particularly involved with masturbation. Owing to certain resemblances to the acute confusional psychoses, it has been suggested that dementia precox is nearly allied to them, and Kraepelin has theorised as to its dependence on a sexually-evolved auto-toxine, apart from the essential intrinsic predisposition. In such very predisposed persons the nerve-cells are highly sensitive to all prejudicial influences. When the enormous changes produced throughout the whole system by the elaboration or deprivation of the sexual function are considered, it certainly seems possible that the more susceptible of the nervous structures in degenerate persons are unable to withstand the intense stimulus of the sexual evolution. This may correspond with some special auto-toxine cast into the circulation (as proposed by Kraepelin) of the nature of a cyto-lysin, producing violent tumult and eventual extinction of function. In other forms of dementia precox it would appear that there is a failure of sufficient energy to continue the development of the individual brain, or even to maintain the level reached. In such cases, too, it cannot be said that the nervous system has succumbed to the stress of combating a hostile environment, but rather that disequilibrium and decay have occurred from inherent weakness of organisation, the nerve-cells and tracts involved simply fading, passing-out of circuit and being replaced by connective tissue. Partly, also, the lack of resistance and active function allows injurious effects to arise from imperfect metabolism and accumulation of waste products around the nerve-cells.

It has always been unfortunate that the literal translation of the German title has been adopted in England instead of a synonym expressing the true German meaning. Kraepelin's syndrome does not fit the nomenclature in its strict English sense, although it is applicable to certain cases such as several

English psychiatrists have described under the head of adolescent insanity and primary dementia. And personally we prefer to keep it restricted to the latter and some irrecoverable cases of the former.

Another important established disorder is amentia or confusional insanity. This now embraces many of the maniacal and agitated melancholic cases formerly classed as delirious; many puerperal cases, acute delirious outbreaks in the degenerate as well as certain partial stupors. It was first separated as a syndrome under the title of Meynert's amentia. In its modern acceptation it comprises the infectious, toxic and exhaustion psychoses. Thus there belong to this category many of the disorders of pregnancy, puerperium and lactation, uræmic deliirum, deliria following on infectious fevers, acute rheumatism, pneumonia, gastro-intestinal toxæmias. It is not, of course, to be assumed that all cases of insanity occurring with puerperal conditions or lactation are necessarily of confusional nature; the aspect will be modified by the relative strength of predisponent and excitant cause, or its genuine toxic origin. There belong, in addition, to amentia states of mental perturbation and confusion resulting from acute or prolonged physical fatigue or primary psychic exhaustion occasioned by some protracted conflict with painful, incessant ideas or delusions. The purely endo- and exo-toxic forms show evidence of a general affection of the peripheral nervous system and the bodily organic functions.

Confusional insanity is not determined by age, and only inconstantly by notable inherited neurosis; patients are often of average or good mental development. Where, however, it appears as the result of hereditary influence the psychoses investigated in the family history are most often found to have been of the same nature, this being largely so because the main exciting causes, pregnancy, puerperium and lactation are peculiar to women, who furnish the larger proportion of confusional cases. Although prodromata are usually apparent, the malady in its grave form is often fulminating and the reduction in consciousness deep. Like the so-called hebephrenic-catatonic stage in dementia precox, there is intense dissociation of faculties, hallucinations and volitional abandonment. There is perception but not realisation of the environment, inferences are confused, there is distressed bewilderment. All mental operations are hesitant or interrupted, even perhaps to the extent of word-mutilation. The strange phantasmagoric world in which the patients dwell inspires them with terror and uncertainty. There is not, however, the wholly irrational resistance, the grotesque gestures and antics, disconnected utterances and attitudinising of the precocious dement; the aspect, though variable, is mainly in conformity with the emotional mood. In milder cases, where inertia and passivity are the main features, the mind of the patient can be observed groping painfully for its appropriate expression, and its attitude, if simple and child-like, is not bizarre or ridiculous. Association of ideas is slow, interrupted; chance annexations are formed. Speech is disjointed, there is neither the rapid casual observation and superficial incoherence of the acute maniac, nor the automatic, spasmodic and wholly unrelational utterances of the precocious dement. With the maniac there is an acute though momentary appreciation of the environment; but such limited and shifting attention as is possible to the ament is concentrated on his interpretations of the confused surroundings. The extravagances of the acute delirious form of confusional insanity are largely due to the insistent but continually varying hallucinations of all senses. Partly from mental exhaustion, in part from physical conditions, there is grave risk of collapse. The contrary state of passivity may be ascribed to the inability to determine any act, or to a real stupor, and this latter is doubtless most serious as indicating a profound poisoning or exhaustion. Yet it may, as a narcotic condition, allow rest and a chance of recuperation. A stupor which is conjoined with negativism, interrupted by automatism and senseless acts, outbursts of laughter, suggestibility, etc., will draw attention to the possibility of dementia precox.

Serious as are the general malaise, denutrition, the futile irritability causing insomnia, and the gastro-intestinal troubles in confusional insanity, there is an even worse outlook as regards recovery in the maintenance or speedy return of apparent bodily health and normal functions without mental improvement. Thus the persistence of menstruation and sustained body-weight, with the progress of an acute amentia, must constitute a grave omen. As a whole amentia is a recoverable form, and not likely to recur, but its most delirious forms are excessively fatal. Owing to the grave reductions in consciousness, the evident intra-psychic dissociation, the sudden onset, the bodily involvement, amentia has, with some justice, been regarded as appropriately classed as a toxic psychosis. The hallucinatory and dissociated mental state has been ascribed by Stoddart to increased synaptic resistance, severance of peripheral and central perception corresponding to that of peripheral from central neurons.

Melancholia and mania, primary forms in the earlier classifications, have been variously elaborated and then distributed. Allotments have been made to the credit of dementia precox, confusional insanity, folie circulaire, recurrent insanity, and thus the original comprehensive grouping has been split up. Eliminating the first two of these, a redistribution of the rest has been proposed by certain psychiatrists, and a syndrome formed which covers all kinds of mania and melancholia, except an involution psychosis (melancholia at the climacteric). Thus it is suggested that one condition or diathesis, the "manic depressive," should embrace the whole range of the affective insanities. Such a diathesis is assumable, but it is not agreed that attacks of pure mania and melancholia may not occasionally happen which are not repeated, or rarely so, and which are not merely stages of dementia precox. Nor, again, is it wholly acceptable that recurrent mania or melancholia, periodic and otherwise, have not an independent existence rather than being merely an exclusively prominent phase in a duplex mental state. Whilst there are many suggestions of one diathesis underlying the whole of the affective maladies, yet it is an extreme position to exclude either acute isolated or recurrent mania and melancholia as sufficiently separate entities. In marked examples the conditions are antithetic clinically and psychologically, notwithstanding the differences may be of degree only. What it is important to recognise is the constitutional or intrinsic character of some of these states in comparison with the relatively accidental or extrinsic nature of others. There are here many grades. In the case of pure mania isolated attacks are undoubtedly infrequent, and evidence of a sufficing extrinsic cause exceptional. Hence whether the attacks be single or repeated, some constitutional predisposition may be inferred as a rule, although the influence of exposure to recurrent causes, like or unlike, but at any rate adequate, must receive attention.

Melancholia is in somewhat different case; it is more prone to isolated appearance, less to repeated attacks of short duration, and it is certainly more influenced by extrinsic causes, and more apt to develop in a disposition naturally in accord than is the case with mania. We may view the isolated attacks of these effective conditions as a mere lapse from the normal, occasioned perhaps by some toxin exogenic as regards the brain, or at any rate by some cause exercising unusual influence, possibly through the medium of the circulation. In the recurrent form the intervals are completely or nearly lucid, the onset of the psychosis is at a mature adult age, there is no evidence of degenerative stigmata, or of an obviously morbid disposition.

The existence of a manic-depressive psychosis has been inferred from the frequent occurrence of a melancholic stadium before an outburst of mania, from the alternations in circular insanity, or the stray appearance of opposite emotional phases in the course of a recurrent disorder; also from the known oscillations in feeling, diurnal, menstrual, sexual, recognised in exaggerated form in the insane. Griesinger alluded to the rebound of mood after melancholia or vice versa, and assumed that mania corresponded to a deeper-seated mental disorder, and Bevan Lewis has laid stress on the difference between mania and melancholia, as being one of degree in the relative reduction in consciousness, the failure in object—and rise in subject—consciousness constituting a common basis. Nevertheless, both from a psychological and clinical aspect there is abundant reason to admit the discrete existence of the two forms of affective disorder, although they may be but stages of the same process dependent on some common but fluctuating condition. The distinctions between typical cases are pointed out by Lewis. In the maniac, buoyant, inconsequent thought, "overaction on lower planes in the intellectual sphere," giving untrammelled freedom and exaltation in feeling. In the melancholic, overaction in the afferent, impressive sphere, with co-existent inertia in the intellectual and expressive one, feeling pent-up and struggling. The rise in subject consciousness which is common to both determines in depression or elation according to the sphere of disorder, and the obstruction or freedom in intellectual operations. In the melancholic painful thought and restricted activity predominate, intensity, persistence and sluggishness prevail; in the maniacal rapidity, transience and turmoil of thought characterise. The former realises sadly his limitations and disabilities, the latter exults in his imagined emancipation. In either case the real intellectual liberty of the sufferer is impaired or lost.

As already stated, some of the old subdivisions of the affective disorders have been distributed amongst fresh syndromes: the agitated forms to confusional, toxic or exhaustive psychoses; the anergic or inhibitory to dementia precox, or the hereditary degenerate class; the senile to involutional decay. The simple form, purely emotional without confusion, and with mere slow and restricted thought, active despondency and ideas of unworthiness, alone remains as a pure variety with some writers. The same deductions and allotments are made in mania. All varieties are probably but different stages or depths of involvement in the same disorder, although perhaps occasioned by distinct causes. An advance towards the recognition of "manic-depressive" psychosis is afforded by recurrent insanity, or repeated, isolated and short attacks, not relapses, with lucid intervals. Its subjects are adults and predisposed, but not degenerate persons; there are often excitant causes, but these are not essential. It is a form in which neurotic heredity shows itself in frequent lapses from stability rather than serious breakdowns, and in which each attack has an isolated character. Although each disorder, melancholic or maniacal, tends to reappear in its own mood, yet interruptions by or transitions to one of opposite kind may occur. Thus is reached gradually the circular psychosis which is the prominent illustration and argument for manic-depressive insanity. It implies not only frequent recurrence of the disorder, but oscillations, in some cases regular, between a high and low level of intellectual involvement. The varieties of folie circulaire, to use the old term, whether double form or serial, are regarded by some as only exaggerated phases of very usual pulsations or "tidal changes," occurring with a certain regularity in the course of any case. Griesinger and other distinguished observers of his time recognised the cyclical form, although it was not separately classified by them. There is indeed no question as to the existence of alternating phases in this condition of mental instability, but with the exception of a comparatively few typical cases, there is irregularity in the order of their occurrence, their relative importance and their periodicity. What one observer would call merely the rebound from a pronounced mania, another might consider the inseparable though minor phase of a circular disorder; what by one would be considered only the irregularities of continuous instability of insane mood, by a second would be classified as folie circulaire without intermission. The influence of hereditary neuroses in this disorder, and the onset at early adult life, is notable; co-fraternal heredity is thrice as often met with as in other forms of insanity, and there is observable a strong similar heredity in parents and children. It is, in fact, a state of persistent mental instability, in which certain periodicities common in the insane are emphasised: youth and strong predisposition being here combined, the alternations of mood are more frequent and marked, also more variable in their relationship. In many instances it will be found difficult or impossible to separate the cases as circular insanity from the preponderating variations in form and mood which are found in the history of the chronic insane. Manic-depressive psychosis, however, does not so much imply an oscillation between two extremes as an indissoluble interdependence. The plea for it is this latent uniformity, not the patent manifestation of the double phase ; in other words, the disposition, not the display. Let it not be imagined that any new psychosis has been discovered, nor that the melancholic and maniacal aspects are as co-equal as the title would express.

In some psychoses the accidental factor appears to have a relatively strong causal influence, and the more the postponement of a primary or isolated attack of insanity towards the period of natural involution the more is an extrinsic agent to be inferred. Disorders of mind referable to traumatism, to coarse brain lesions either infantile and independent of cerebral development or of later acquisition, to toxic states, such as alcoholism, syphilis, uræmia, with perhaps secondary toxæmias from perverted metabolism—all these are naturally to be ranged at the opposite end of the causation table to those psychopathies which originate in the faulty mental constitution of the patient. The evident importance of many extrinsic factors has stimulated investigation into the somatic states in insanity. Thus, taking Kraepelin's syndrome, dementia precox, we find records of cytological changes in blood and cerebro-spinal fluid, destruction of nucleo-proteids in cerebral tissues, diminution of neutral sulphur, indicating lessened cell-activity and oxidation, imperfections in circulatory system, etc., findings often contested no doubt, but showing the range of research pursued in this and other forms of mental disorder. There is, too, a steady accumulation of valuable and reliable histological data.

More progress has been made in the nature of general paralysis than of any other disorders. Although it cannot be said with assurance that general paralysis is impossible without syphilis, there is no doubt as to the paramount influence of the latter. It is well recognised that there is a distinction between syphilitic dementia with its accompanying lesions and the meta-syphilitic conditions in general paralysis (to use the only practicable term at present). The connecting-link between tertiary syphilis and general paralysis is therefore not yet found. Clinically, it may be said that brain syphilis affords symptoms more erratic and episodic than in general paralysis and of a certain pathognomonic kind, such as severe nocturnal headache, insomnia, paralysis of cranial nerves, mono- hemi- or para-plegias, clonus, Babinski's reflex, amnesia, aphasia, etc., and especially complete iridoplegia. The invariable presence of tender spots found on skull-pressure in cortical syphilis is avowed to be absent in general paralysis. In syphilitics there appears to be less change in character and extinction of personality, more realisation of the mental transformation. Nevertheless, despite subtleties of diagnosis, it will be sometimes hazardous to differentiate between cases of syphilitic insanity, alcoholic insanity, and general paralysis, so far as clinical symptoms are to be relied upon. The presence of a positive Wassermann reaction in the serum merely proves the existence of syphilis, whilst, again a negative reaction in the cerebro-spinal fluid, even with some leucocytosis, only indicates that as yet nervous complications are absent, or if present, are of the nature of a gummatous meningitis. A negative reaction in both cerebro-spinal fluid and serum, again, has been obtained by accurate observers in undoubted general paralysis. But two facts are prominent as regards the relation of the Wassermann reaction to general paralysis. It is rarely met with in insane cases other than general paralysis (excluding those giving evidence of syphilis), and a positive reaction in serum and cerebro-spinal fluid repeated more than once is almost certain evidence of general paralysis. An increased globulin and protein content in cerebro-spinal fluid is found with both syphilis and meta-syphilis, as also lymphocytosis, but the difference in relative amounts is conspicuous.

There may be factors other than syphilis which are capable of producing cortical lesions of similar kind and distribution to those occurring in general paralysis, and hence symptoms in accord. These too, though far less frequently, may lead to an altered metabolism, or the formation of an auto-toxin, which can disturb the functions of the brain cells. It has been theorised that the cortex cells in general paralysis in their attempts to neutralise antigen and in the production to excess of antibody, exhaust themselves and become absorbed. They are destroyed, as it were, in their defensive efforts to supply a surplus of antibodies.

When we regard the part played by other presumed toxic causes in insanity we are upon uncertain ground. The deliria of infective fevers, of certain poisons—alcoholic, alkaloidal, organic, auto-toxic, etc.—have suggested certain extended possibilities in somatic origins. The only condition of actual insanity, however, which is really comparable to the delirium of the infectious fevers, is that of acute amentia or delirious mania. Even this is inferred, but not yet proven to be due to a toxæmia. Less marked conditions can so far only be placed vaguely to the account of a perverted metabolism. A confirmation of the theory of toxæmia has been suggested by the leucocytosis met with in acute phases of mania and melancholia or in exacerbations during their course, also by a relative increase in the neutrophiles. Such are regarded as signs of a reactive protection like to that occurring in abscess, furunculosis, pneumonia, etc. Whilst, again, the marked remissions, even in long-standing cases, which have been caused by chance infections, are perhaps associated with a hyper-leucocytosis, so, on the contrary, the absence of leucocytosis, even leucopenia and diminished neutrophiler percentage, has allowed the inference that toxæmia has prevailed over resistance and an unfavourable forecast in regard to a demential ending to be made. At present there is no settled opinion as to the part played by organisms found in the tissues of the insane; but most observers consider their presence as casual, or as ante-mortem invasions; at most they may contribute secondary symptoms. It is seldom in any case that organisms themselves are found in or near the nerve-cells.

That the opsonic indices of blood in the insane are generally lowered slightly, though notably in the acute forms, does not infer more than a lowered resistance perhaps, but not of necessity, directly related to mental disorder. Many other investigations might be mentioned, but it is here unnecessary to do more than show the attempts being made to place insanity on the same level as other disorders. Even so far, in addition to the recognition of the part played by hereditary predisposition and actual degeneration, that of the causal influence of presumed toxic and infective factors, of traumatism, gross lesions and so forth has immensely improved schemes of classification by giving relative weight to the internal and external agents bearing on the production of insanity. This balance must always have considerable value in forming a prognosis. Consequently the best classification must be based on causation; at one end of the table being placed those conditions in which predisposition seems to have least influence and causes external to the brain the most, and at the other end those states in which there is most constitutional proclivity to psychopathy, and accidental causes are of slight or no contributory value.

Such cases as might fall within the first group would comprise the cerebral lesions of infant or adult life not dependent on any developmental fault, but due to injuries, infections, arterial diseases, hemorrhage, etc., where the external cause seemed to be solely or mainly operative. A group in which there is a varying influence of accidental and constitutional causes will be formed by the so-called toxic and infectious maladies, amongst which are alcoholism, syphilis, thyroidism, influenza, metabolic perversions, and so on. In all these a certain predisposition is required for the production of the more definite psychoses, although states of confusion, delirium and dementia may be induced apart from this. Of the affective disorders, the occurrence of which in pure isolated attacks will be rare, melancholia, more elicited by external causes, may be placed at one end of the group, and mania, for which an extensive cause is uncommon and a constitutional one likely, at the other end. Possibly in persons of active imagination, great sensitiveness and finely-balanced faculties, these attacks may be produced as a result of shock, prolonged mental or bodily strain, of severe self-repression, over-excitement or reaction from deep grief, apart from any discoverable predisposition.

In the sparsely recurrent or relapsing attacks of affective insanity, constitutional tendency becomes more definitely the important factor, but there may yet be found a determining influence external to the brain or to the person ; but the frequently recurrent form, alternating, irregular, or repeating, indicates a special excitability and inherent defect of brain, a state of persistent mental instability, in which the need for an extrinsic cause is scarcely obvious.

Following these are the involutional psychoses, in which the mental disorder coincides with the decay of sexual life, or with anticipated senile changes, associated or not with some influential somatic condition.

The adolescent insanities, including dementia precox, approach the class of developmental defectives, inasmuch as the actual breakdown, or even the supposed contributory cause, is the result of the evolutionary anomaly. The serious disintegration of mind, acute progress, co-existence in other members of the family, and frequent irrecoverability, place the whole group in the category of those predestined to insanity Whilst some members of this are said to recover permanently, most of the less unfavourable cases will remain in the relapsing or persistently unstable class. We cannot, however, entirely eliminate some added toxic agent, or an undue susceptibility to toxins not necessarily abnormal in themselves. Almost last amongst the insane states are the true degenerative diatheses, persistent and irremediable, upon which acute or chronic deliria may be grafted, and which may include constitutional immorality, sexual perversions, congenital weakness, states of defective control, general neurasthenia and hysteria. This group, whilst showing congenital and irreparable vices of organisation, is in part not prone to any precocious demential ending, in part so far as the engrafted delirious forms are concerned, so notably prone.

Finally the class of arrested developments from inherent causes is reached. Thus briefly and imperfectly is outlined the principle underlying a classification, which in its plan promises to endure, the placing of the particular disorder being but a matter of the knowledge of its nature, origin, and relation to other mental conditions already having a defined position in the scale.

---

### THE RIGHT AND THE WRONG SIDE OF THE X-RAY PICTURE; HAS A MISTAKE BEEN MADE?[1]

